# Hemi-tibia allograft and free microvascularized fibula transplant reconstitute the tibia shaft with side to side healing: 7 year follow up of a 14-year-old boy with adamantinoma

**DOI:** 10.1080/23320885.2021.1999246

**Published:** 2021-11-11

**Authors:** Alexander P. Decilveo, Melissa S. Liebling, Andrew L. Golden, James C. Wittig

**Affiliations:** a Seton Hall, St. Joseph’s University Medical Center, Paterson, NJ, USA; b Hackensack University Medical Center, Hackensack, NJ, USA; c Morristown Medical Center, Morristown, NJ, USA

**Keywords:** Adamantinoma, osteofibrous dysplasia, free microvascularized fibula, hemi-tibia allograft

## Abstract

Adamantinoma is a malignant tumor that usually presents in adult men between 20 and 50 years. Due to its metastatic potential, differentiating Adamantinoma from Osteofibrous dysplasia is essential as treatment varies greatly. We present a case of limb salvage using a free microvascularized fibula transplant and hemi-tibia allograft.

## Introduction

Adamantinoma is a low-grade malignant tumor with epithelial differentiation and a marked predilection for the tibia [1,2]. Classically, plain radiographs demonstrate an expansile lesion with a soap bubble appearance involving the tibial diaphysis and diffuse hyperintensity on T2 weighted imaging with periosteal thinning. Histologically, this tumor can be composed of four different cell type patterns, but always with epithelial differentiation similar to Osteofibrous dysplasia (OFD). A stepwise diagnostic approach involving an orthopedic oncologist, musculoskeletal radiologist and pathologist are key to an accurate diagnosis. Given OFD is a benign entity, differentiating these tumors is imperative as treatment varies from local excision to radical resection. Options for reconstruction following radical resection include endoprosthetic implants, free microvascularized fibula autograft, allograft, and distraction osteogenesis. Surgeon preference dictates reconstruction modality. In this paper, we present our personal experience using a free microvascularized fibula transplant with extended clinical and radiographic follow-up.

## Case report

A 14-year-old, otherwise healthy boy presented to his pediatrician with 2 weeks of painless swelling in the right leg. He was referred to an orthopedic oncologist and underwent an X-Ray and MRI. Physical examination revealed a slightly palpable, immobile, and convex curvature of his right tibia. There was no history of fevers, chills, trauma, numbness, or tingling. No significant medical history. Family history included colon cancer and prostate cancer.

**Figure 1.**
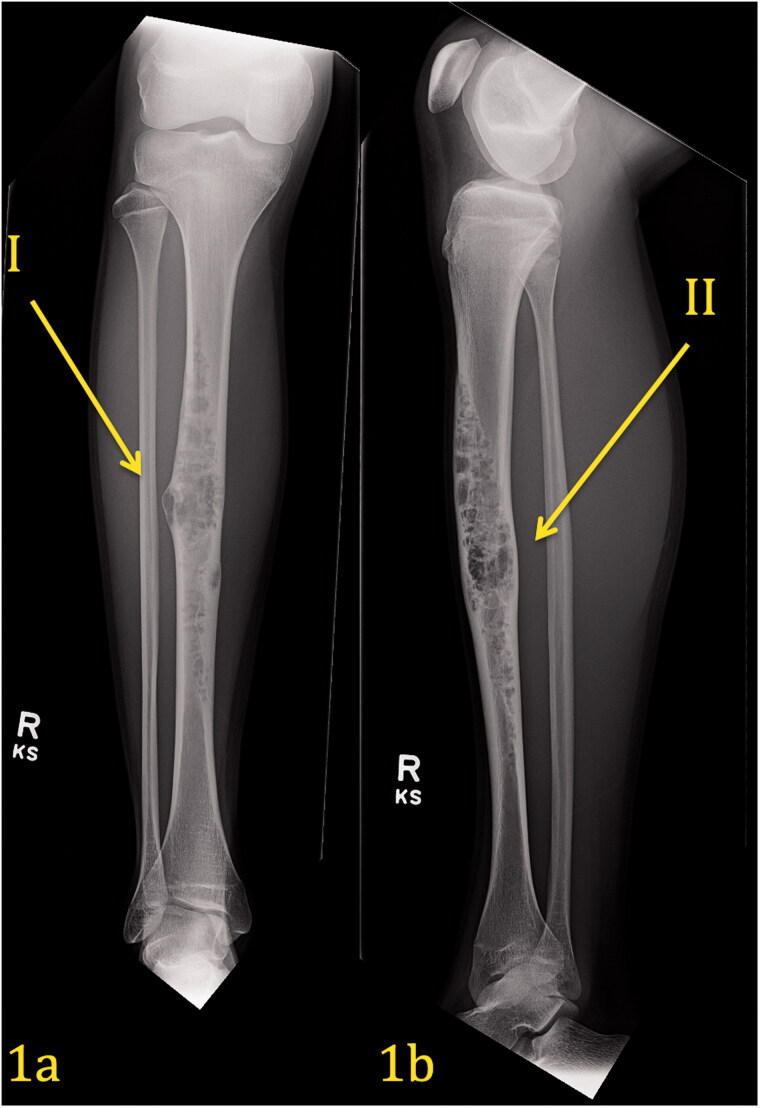
(a,b) Pre-operative Imaging Right Tibia. AP view (1a) of the right leg demonstrates an expansile, multilocular, lytic lesion with ‘soap bubble’ appearance and narrow zone of transition within the diaphysis of the tibia affecting the cortex and medullary canal. There is associated cortical remodeling and endosteal thinning. No cortical breakthrough is present and only a slight benign-appearing periosteal reaction is present (Arrow I). Lateral view (1b) of the right leg corroborates the findings on the frontal projection.

**Figure 2.**
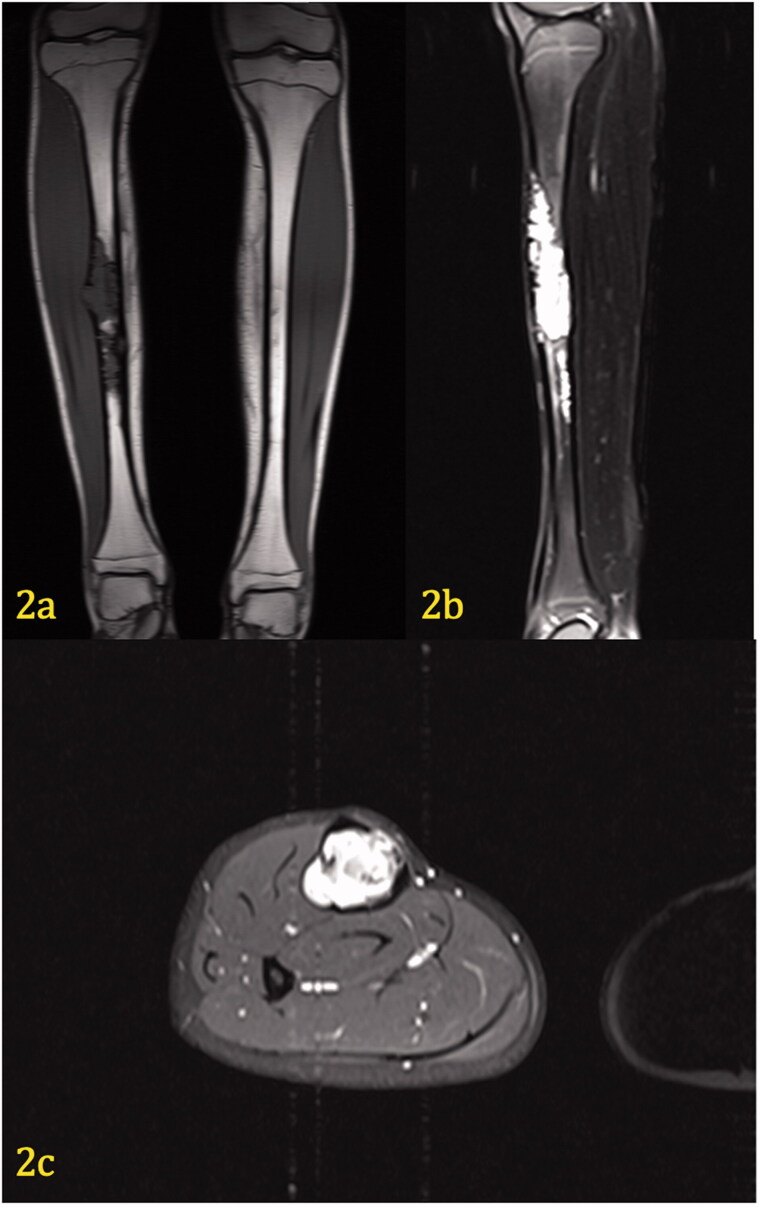
(a–c) Coronal T1 weighted image (2a) demonstrates hypodensity of a tibial lesion. Sagittal fat-saturated T2 weighted image (2b) of the left tibia demonstrates marked hyperintensity of the lesion, which extends from the anterior to posterior cortex. Axial contrast-enhanced, fat-saturated T1 image (2c) through the mid diaphysis demonstrates marked enhancement of the soft tissue mass, which completely obliterates the marrow. The cortex is thinned, but there is no extension of mass beyond the periosteum. No edema is present within the musculature.

Laboratory tests that included complete blood count, chemistries, prothrombin time/partial thromboplastin, were normal. Imaging studies, AP/lateral X-rays of the right tibia, MRI of the right tibia with and without contrast (Figures 1(a,b) and 2(a–c)) were performed. A biopsy was subsequently performed.

## Imaging interpretation

Figure 1(a,b) demonstrates a pre-operative plain radiograph of the right tibia and fibula. An expansile lytic lesion with a soap bubble/moth-eaten appearance can be seen within the diaphysis of the tibia. The lesion extends throughout the midshaft in all dimensions of the tibia. It involves the cortex as well as the medullary canal. Endosteal thinning is noticed along the lateral and medial cortex. Sclerotic margins are present surrounding other areas of the lesion. There is no evidence of a periosteal reaction, but the lesion is expansile, with thinning of the lateral cortex. The tibia appears slightly bowed.

On T-2 fat-saturated MR images, the lesion is hyperintense to muscle and fat (Figure 2(a–c)). In the sagittal view, the proximal portion of the lesion extends from the anterior tibial cortex to the posterior tibial cortex. The lesion is approximately 19.2 cm in length. Axial contrast-enhanced images demonstrate cortical thinning along the medial aspect of the midshaft of the right tibia. A very slight periosteal reaction can be seen laterally and medially. The tumor transgresses the cortex laterally in a small area, however, the periosteum appears intact around this area and the expansile areas. There is no evidence of soft tissue involvement or a hemorrhagic component.

## Histology interpretation

**Figure 3.**
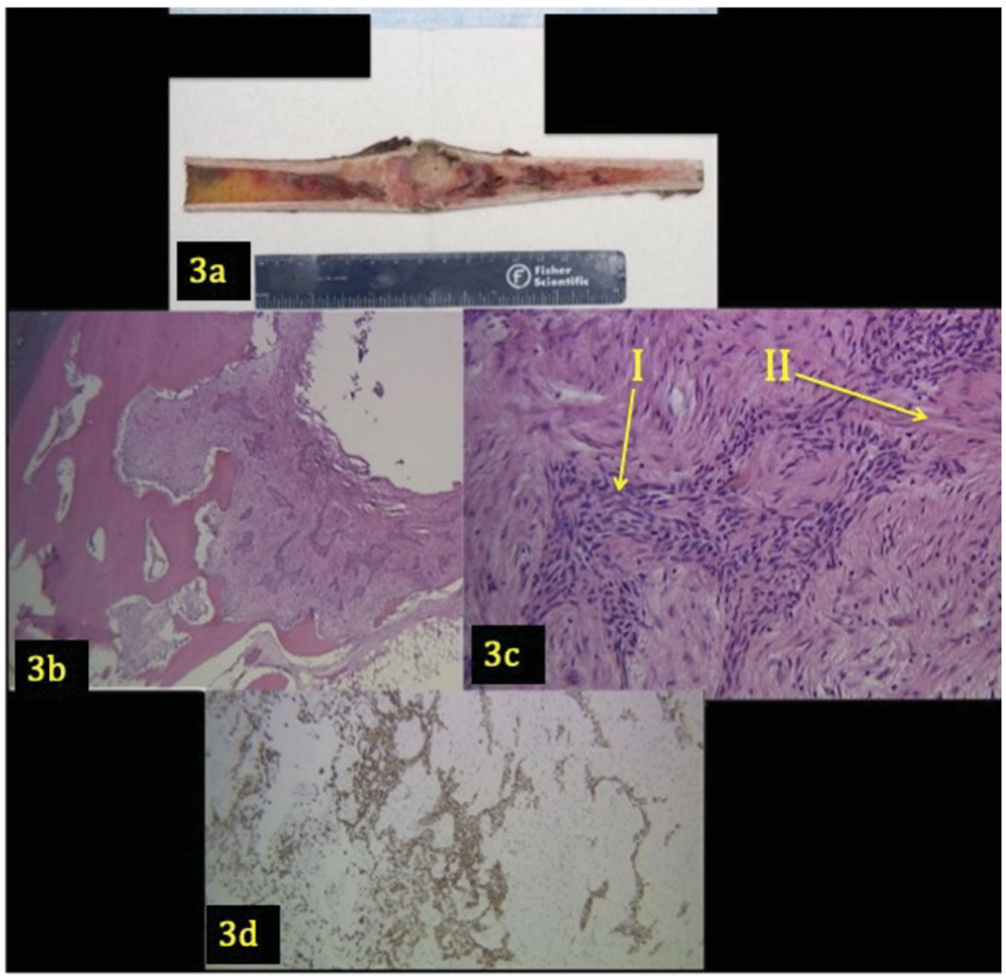
(a–d) Gross image and pathology of tibial lesion. A longitudinal section shows a 9.2 × 3.6 × 2.7 cm intramedullary tumor that involves the cortex (3a). Low power (3b) and high power (3c) views demonstrate a lesion with a biphasic pattern characterized by epithelial islands (Arrow I) surrounded by an abundant fibrous stroma (Arrow II). Figure 3(d) shows the epithelial component demonstrating positivity for pan keratin.

An open biopsy of the patient’s right tibial shaft was analyzed (Figure 3(a–d)). Grossly, a longitudinal section revealed an intramedullary tumor that involved the cortex (Figure 3(a)). Microscopically, the lesion demonstrates a biphasic pattern characterized by epithelial islands surrounded by abundant fibrous spindle cells. The cells demonstrated mild pleomorphism, but no mitoses or necrosis were evident. The epithelial component was confirmed with a pan keratin immunohistochemical stain that spares the fibrous stroma. The epithelial cells were CD31 negative.

## Surgical procedure

The patient was treated with radical resection of the right tibia shaft and reconstructed with a hemi-tibia shaft fresh-frozen allograft and free microvascular fibula transplant. Through a medial approach to the leg, a total of 19 cm of the tibia with 2 cm margins at the proximal and distal end was removed. A free vascularized fibula was harvested from the contralateral limb in a manner that its length would permit direct contact or overlap with the tibia proximally and distally. The graft was prepared with preservation of the peroneal vessels, which were later anastomosed to proximal branches of the posterior tibial artery. No skin island was used as an implanted pulse oximeter ensured patency of the anastomosis. The allograft used was a fresh frozen tibia that approximated the patient’s size (Bone Bank Allografts, San Antonio, TX). The tibia allograft was then cut longitudinally approximately 50% of its circumference to enable space for the free vascularized fibula to be recessed. The hemi-tibia allograft was fixated to the native bone with two pelvic reconstruction plates that were contoured for placement. The fibular autograft was lagged to native bone distally and allograft proximally. The contact between the free fibula and native tibia as well as the space between fibula and allograft were supplemented with a mixture of iliac crest autograft and cortico-cancellous bone allograft.

## Discussion

In this case, a 14-year-old remarkably healthy boy was seen by his pediatrician for a painless mass in the diaphysis of his right tibia that proved to be an adamantinoma. The young age of the patient is atypical for developing an adamantinoma, however, the anatomical location along the anterior tibial diaphysis with extensive intramedullary involvement and cortical thinning, bowing of the tibia, and histological studies demonstrating a biphasic neoplasm with strong epithelial differentiation and spindle cells are consistent with the diagnosis of adamantinoma.

Adamantinoma is a low-grade malignant tumor with epithelial differentiation and a marked predilection to be located in the tibia. This tumor resembles the more prevalent ameloblastoma of the jawbone. Adamantinoma usually affects adolescents and young adults, however, a study done by Moon and Mori reports a mean age of 32.9 years [1,2]. Symptoms include swelling and local pain; 33% of patients have had symptoms for longer than five years [1,3].

While 85% of cases involve the tibia, there are reports in the literature involving all long bones [1]. When bones other than the tibia and fibula are involved, unusual histological features have been noted [4]. Histologically, this tumor can be composed of four different cell type patterns, but always with epithelial differentiation. The four patterns are basaloid, spindle, tubular, and squamous. Our case most closely resembles the spindle pattern: small, uniform spindle cells with the presence of clefts surrounded by epithelial cells [5]. As stated before, adamantinomas demonstrate cytokeratin positivity. Adamantinoma may be misdiagnosed as osteofibrous dysplasia (OFD) because of their similarities. Czerniak et al. separated adamantinoma into two classes: a classic and differentiated type [6]. The classic type has most of the features of OFD with the presence of epithelial cells, as in our case [5]. The differentiated type is characterized by an OFD pattern with scattered positivity of cytokeratin for epithelial cells [6].

The main differential diagnosis in this patient is between OFD and adamantinoma. Both of these tumors arise from the tibia. Osteofibrous dysplasia is a benign fibro-osseous lesion of bone that may prelude or regress into adamantinoma. OFD most commonly affects patients in the first two decades of life, rarely affecting them after skeletal maturity. It usually arises in the cortical bone of the anterior mid-shaft of the tibia [7]. Clinically, this slow-growing lesion most frequently presents with symptoms of swelling and mild discomfort as well as bowing of the tibia. Radiographically, OFD is associated with thinning and expansion of the anterior cortex, sclerotic rimming, and a ‘soap bubble’ appearance. These lesions do not typically have a periosteal reaction or fill the medullary canal as did the adamantinoma in this case. MRIs are rarely useful for differentiating OFD from adamantinoma as both can be heterogeneous on T-1 and hyperintense on T-2 weighted sequences.

OFD can usually be observed, and if symptomatic, curetted and bone grafted. Due to the high recurrence rate of adamantinoma when treated with curettage, wide resection with a prosthesis or some form of the structural bone graft should be considered. Chemotherapy and radiation are not considered effective [8].

**Figure 4.**
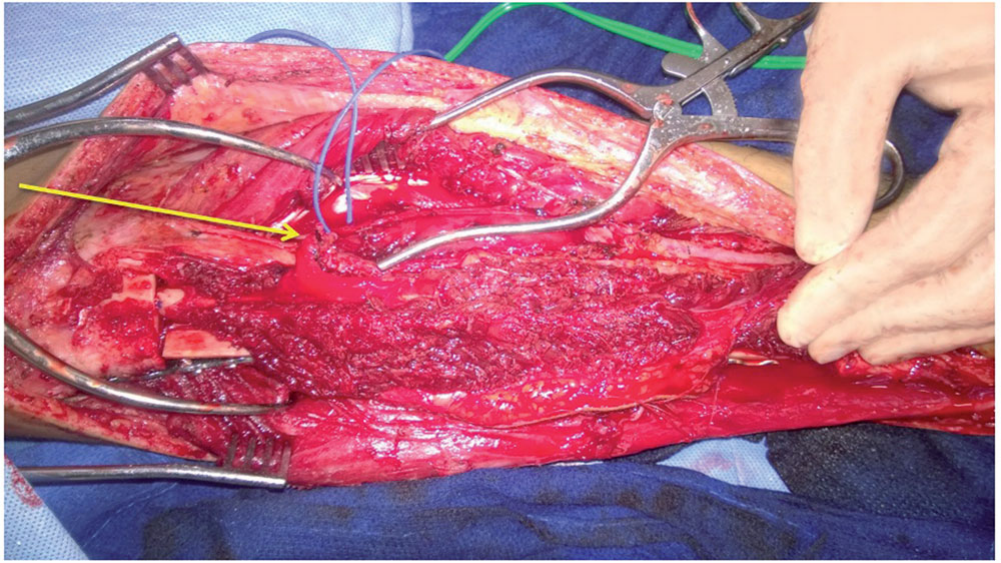
Intra-operative image of free microvascular fibular graft. Unconnected vascular structures can be seen in the free microvascular fibular graft (Arrow I). Beneath the transferred fibula, the hemi-tibia allograft plated with screws to the proximal portion of the tibia can be seen.

**Figure 5.**
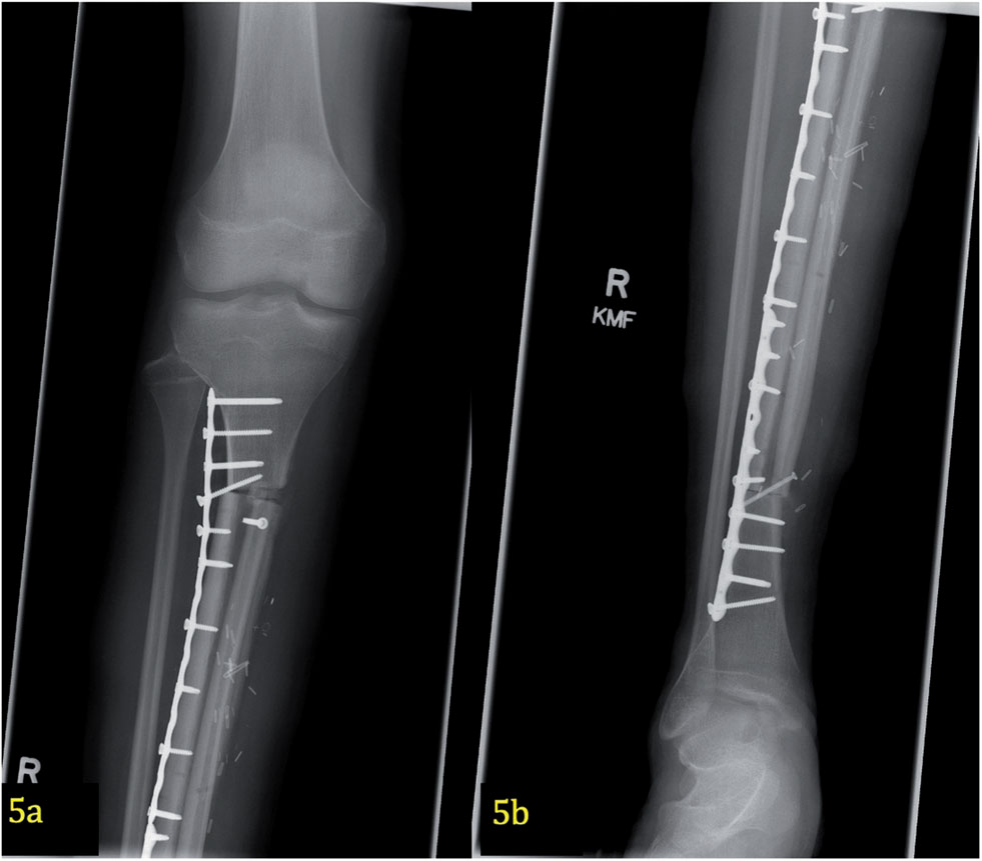
(a,b) Post-operative X-ray of free microvascular fibular graft. At 29 months postoperatively, proper alignment of the hemi-tibia allograft and free microvascular fibular graft can be seen in the proximal (5a) and distal (5b) portions of the tibia. This AP image shows two plates with screws spanning the entire defect.

**Figure 6.**
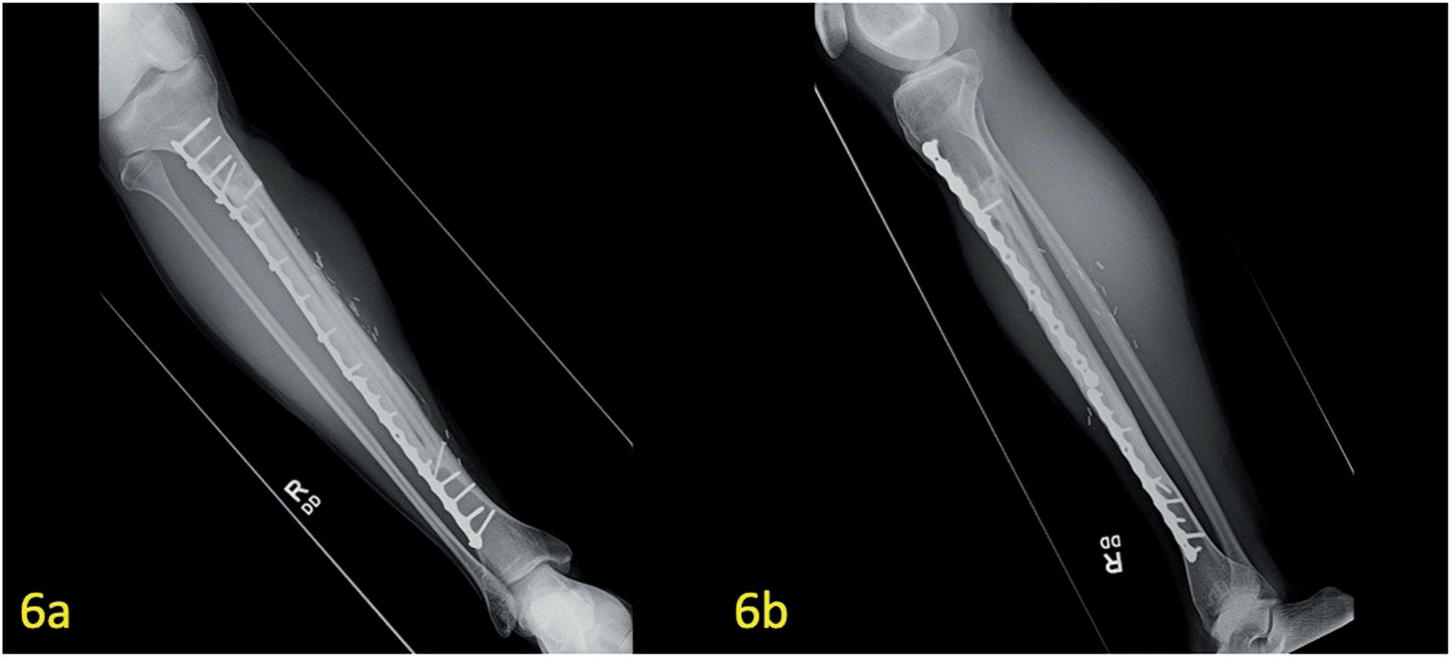
(a,b) Post-operative X-ray of free microvascular fibular graft at 7 years postoperatively. Union of the hemi-tibia allograft and free microvascular fibular graft with proper alignment of hardware can be seen on the AP/lateral radiographs.

The patient was treated with radical resection of the right tibia and reconstruction with a hemi-tibia fresh-frozen allograft and free microvascular fibula transplant. An intra-operative photograph demonstrates the peroneal vessels from the newly transferred microvascular fibular graft (Figure 4). Hypertrophy of the microvascular fibular graft was achieved, and this result has been associated with positive outcomes [9]. The tibia allograft, which was cut longitudinally, offers additional structural support to the transferred fibula until the fibula can heal and hypertrophy. Radiographs 2 years postoperatively demonstrate good healing of the allograft and fibula. The fibula has hypertrophied (Figure 5(a,b)). At 7 years postoperatively, adequate union and incorporation of the graft into the native bone and allograft is seen (Figure 6(a,b)). There have been no complications. The patient is currently ambulating without pain, participating in sports and has normal knee function.

Other options for reconstruction include intercalary prosthesis, pure allograft, distraction osteogenesis, and masqualet procedures. The choice of a hemi-tibia allograft and free microvascular fibula transfer was preferred over prosthesis or complete allograft reconstruction due to low rates of infection, fracture, non-union, and hardware failure [9,10]. Given the patient’s age, the senior surgeon’s goal was to preserve both adjacent joints and provide the patient a single-stage procedure that would heal reliably. With graft hypertrophy into native bone, our aim was to provide this patient with as close to a normal functioning limb lifetime. While intercalary prosthesis offers a viable option of immediate fixation and weight-bearing, the patient would have activity limitations in running and contact sports. Regarding distraction osteogenesis and masqualet procedures, there is a paucity of studies in the oncological literature with most of the research limited to case reports and small case series [11–13]. Given the concern for tumor activation with distraction osteogenesis, although theoretical, we opted for a method of fixation that required a single procedure without concern for a surge of growth factors and potential reactivation of tumor cells. This method for exceptionally large defects requires frequent follow-up and carries a risk of pin loosening, pin tract infection and deep bone infection [11–13]. While the aforementioned options of reconstruction are appropriate for this patient, further studies directly comparing these methods directly are necessary to fully evaluate the optimal method of fixation for bony defects following tumor resection in skeletally immature patients.

## Ethical approval

Each author certifies that his or her institution approved the reporting of this case report and that all investigations were conducted in conformity with ethical principles of research.
